# Schrödinger–Poisson systems under gradient fields

**DOI:** 10.1038/s41598-022-20107-9

**Published:** 2022-09-20

**Authors:** Kamel Ourabah

**Affiliations:** Theoretical Physics Laboratory, Faculty of Physics, University of Bab-Ezzouar, USTHB, Boite Postale 32, 16111 El Alia, Algiers Algeria

**Keywords:** Ultracold gases, Plasma physics, Theoretical physics

## Abstract

A singularity-free generalisation of Newtonian gravity can be constructed (Lazar in Phys Rev D 102:096002, 2020) within the framework of gradient field theory. This procedure offers a straightforward regularisation of Newtonian gravity and remains equally well applicable to other fields, such as electromagnetic fields. Here, with the aim of finding potentially measurable effects of gradient fields on the dispersion properties of various media, we present a quantum kinetic treatment of matter under gradient fields. The method is based on the application of the Wigner–Moyal procedure to the modified Schrödinger–Poisson equation emerging in the framework of gradient field theory. This allows us to treat, on equal footing, three different scenarios, namely self-gravitating systems, plasmas, and cold atoms in magneto-optical traps. We address the signature of gradient fields in the elementary excitations of these media. In particular, we estimate this effect to be accessible in state-of-the-art plasma-based experiments. We discuss in detail the classical kinetic and hydrodynamic limits of our approach and obtain a class of generalised Lane–Emden equations, in the context of gradient field theory, which remain valid in the three scenarios discussed here.

## Introduction

The important role of *analogies* has been demonstrated by many examples in the history of science as they permit ideas from different realms of science to be applied in other fields. Arguably, the most studied analogies in modern physics concern analogue models of gravity which attempt to model various phenomena of gravitation using different physical systems, such as dielectric media, (super)fluids, Bose–Einstein condensates, etc. The motivation for studying such formal analogies, and unveiling their origins, is that these analogues may create new “and sometimes unexpected” avenues for practical experiments within the analogue that can be applied back to the source phenomenon. The concept of a condensed-matter gravity analogue was first considered in a little-known paper by Gordon^1^, who noticed that moving isotropic media appear to electromagnetic fields as certain effective space-time geometries. Since then, several other analogue systems have been studied as possible platforms for emulating both general relativity^2–4^ and Newtonian gravity^5–7^ phenomena.

Here, we wish to exploit these analogies in the context of gradient field theory. The latter presents itself as a promising way to regularise the (gravitational or electromagnetic) fields, resulting in a theory free of singularities. In fact, the singularities in Newtonian gravity indicate the limits of applicability of the theory; a feature that has motivated a growing interest in recent years for studying small-scale generalisations of (and deviations from) Newtonian gravity^8–10^. The same is true for classical Maxwell electrodynamics, which has led to the introduction of various regularisation schemes; the most known being the so-called nonlinear electrodynamics of Born^11^ and of Born and Infeld^12^. An even simpler and easy-to-use alternative is provided by the formalism of gradient field theory. The concept of (second) gradient Newtonian gravity was thoroughly discussed recently by Lazar^13^, while gradient field electrodynamics has a longer history; it has been studied for first gradient fields (viz. Bopp–Podolsky electrodynamics) in^14–16^ and for second gradient fields in^17^. This procedure appears very promising as it allows for a straightforward regularisation of the fields. To date, however, an experimental test is still lacking and appears to be extremely challenging given the difficulty of probing such a small-scale effect^13^. In light of that, it may be relevant to change perspective and seek possible imprints on the dispersion properties of various media. This paper attempts to explore this possibility.

More precisely, we shall consider a class of systems that can be formally described by the Schrödinger–Poisson (SP) equation [also known as the Schrödinger–Newton equation]. The latter results from the combination of the Schrödinger equation and the Poisson equation describing the self-potential. Historically speaking, the SP equation was first advocated in the context of gravitation by Diósi^18^ and Penrose^19^ as a simple quasi-classical model to introduce quantum effects in gravitational problems. It describes quantum matter confined by gravitational fields and, as such, finds many applications in astrophysics; it describes (hypothesized) boson stars^20,21^ (which could be a source of ‘exotic’ Laser Interferometer Gravitational-Wave Observatory (LIGO) detections^22^) and it is a central ingredient of scalar field dark matter models^23–26^. Interestingly, the same equation applies equally well to a variety of other systems, such as quantum plasmas^27–29^ and atomic gases in magneto-optical traps (MOTs)^30–32^. A bridge with Bose–Einstein condensates can also be established^33–35^ (see also^36^ for a more general discussion). These similarities may inspire future laboratory experiments using gravity analogs.

Here, we are interested in the gradient field extension of the SP equation. By applying the so-called Wigner–Moyal^37,38^ procedure, we develop a kinetic treatment of the SP equation under gradient fields. Such a treatment allows studying the collective behavior of systems of *N* self-interacting quantum particles, where the self-interaction formally obeys the Poisson equation. It covers gravitational systems^23–26^, quantum plasmas^27–29,39^, and cold atoms in MOTs^30–32^. This allows us to reveal the “fingerprints” of gradient fields in the dispersion properties of these media and the corresponding elementary excitations, i.e., Jeans oscillations/instability in the case of a self-gravitating system (SGS), electron states or plasmons in the case of a quantum plasma, and hybrid oscillations in the case of MOTs. We estimate this effect to be accessible in present plasma-based experiments.

The paper progresses in the following fashion. For completeness, we lay out in “Gradient fields and Poisson-like equations” the theoretical background of gradient field theory and present the resulting Poisson equation. In “Schrödinger–Poisson systems for gradient fields”, we derive and discuss the corresponding SP equation. In “Quantum kinetic approach”, we present the quantum kinetic treatment of the SP equation and analyze the corresponding dispersion relations. In “Classical limit: the Vlasov–Poisson regime”, we analyze in more depth the classical kinetic limit of this approach, i.e., the Vlasov regime. In “Hydrodynamics and generalised Lane–Emden equations”, we present the hydrodynamic limit of this model and obtain a class of generalized Lane–Emden equations that remain valid in the three scenarios discussed here. We present our conclusions in “Conclusion and outlook”. For simplicity, we restrict the discussion in the main text to first gradient fields, involving a single internal length scale parameter. Second gradient fields (involving two internal length scale parameters) can be studied following similar lines of reasoning, at the cost of a more complex formalism, and will be discussed in the supplementary material.

## Gradient fields and Poisson-like equations

We provide in this section the field-theoretical framework of (second) gradient modification of Newtonian gravity and Maxwell classical electrodynamics (more details may be found in Refs.^13,17^). We also discuss a bridge that can be established with atomic gases in MOTs.

The Lagrangian density for second gradient modification of Newtonian gravity reads as^13^

1 $$\begin{aligned} \mathscr{L}=\frac{1}{8 \pi G}\left( \varvec{g} \cdot \varvec{g}+\ell _{1}^{2} \nabla \varvec{g}: \nabla \varvec{g}+\ell _{2}^{4} \nabla \nabla \varvec{g} \vdots \nabla \nabla \varvec{g}\right) +\rho \Phi , \end{aligned}$$

where $\Phi$, $\mathbf {g}$, and $\rho$ denote, respectively, the gravitational potential, the gravitational field strength vector, and the mass density, while $\nabla$ denotes the del operator. In Eq. (1), we have the notations $\varvec{g} \cdot \varvec{g}=g_{i} g_{i}$, $\nabla \varvec{g}: \nabla \varvec{g}=\partial _{j} g_{i} \partial _{j} g_{i}$, and $\nabla \nabla g \vdots \nabla \nabla g=\partial _{k} \partial _{j} g_{i} \partial _{k} \partial _{j} g_{i}$. The parameters $\ell _1$ and $\ell _2$ are two internal characteristic length scale parameters, whose values might be determined experimentally. From the Lagrangian density (1), the Euler–Lagrange equation follows as

2 $$\begin{aligned} \begin{gathered} \frac{\delta \mathscr{L}}{\delta \Phi } \equiv \frac{\partial \mathscr{L}}{\partial \Phi }-\nabla \cdot \frac{\partial \mathscr{L}}{\partial (\nabla \Phi )}+\nabla \nabla : \frac{\partial \mathscr{L}}{\partial (\nabla \nabla \Phi )} -\nabla \nabla \nabla \vdots \frac{\partial \mathscr{L}}{\partial (\nabla \nabla \nabla \Phi )}=0. \end{gathered} \end{aligned}$$

The latter produces a modified Poisson equation for the gravitational potential $\Phi$ that reads as^13^

3 $$\begin{aligned} L(\Delta ) \Delta \Phi =4 \pi G m n(\mathbf {r}), \end{aligned}$$

with

4 $$\begin{aligned} L(\Delta )=1-\ell _{1}^{2} \Delta +\ell _{2}^{4} \Delta ^{2}, \end{aligned}$$

where *m* is the mass, $n(\mathbf {r})$ is the number density and $\Delta$ denotes the Laplacian. Similarly, in the case of second gradient electrodynamics, one arrives at the following generalized Poisson equation for the scalar potential $\phi$^17^

5 $$\begin{aligned} L(\square ) \square \phi =\frac{Q}{\varepsilon _{0}} n(\mathbf {r}), \end{aligned}$$

where *Q* and $\varepsilon _0$ denote, respectively, the electric charge and the permittivity of vacuum, and

6 $$\begin{aligned} L(\square )=1+\ell _{1}^{2} \square +\ell _{2}^{4} \square ^{2}, \end{aligned}$$

with

7 $$\begin{aligned} \square :=\frac{1}{c^{2}} \partial _{t t}-\Delta \end{aligned}$$

being the d’Alembertian. We will restrict ourselves here to the non-relativistic regime which can be formally obtained by setting $c \rightarrow \infty$. In this case, Eq. (5) reduces to

8 $$\begin{aligned} L(\Delta ) \Delta \phi =-\frac{Q}{\varepsilon _{0}} n(\mathbf {r}). \end{aligned}$$

The modified Poisson equations (3) and (8) can be used, respectively, to describe a system of particles confined by gravitational forces or a plasma where the species interact via electrostatic interactions. Other scenarios, formally obeying the same class of equations, can also be considered in relation with cold atom physics. In fact, in the case of an atomic cloud confined and cooled in a MOT, two main forces emerge, which formally obey a Poisson-like equation; hence the regularization scheme provided by gradient field theory applies in this case as well. First, one has the *absorption force* $\mathbf {F}_A$, associated with the gradient of the incident laser intensity due to laser absorption by the atomic cloud. It is an attractive force which can be determined by a Poisson type of equation as^40^

9 $$\begin{aligned} \mathbf {\nabla } \cdot \mathbf {F}_{A}=-\frac{\sigma _{L}^{2} I_{0}}{c} n(\mathbf {r}), \end{aligned}$$

where $\sigma _L$, $I_0$ and *c* denote, respectively, the laser absorption cross section, the intensity of laser beams, and the speed of light. In addition to the absorption force $\mathbf {F}_{A}$, one has the *radiation trapping force* $\mathbf {F}_{R}$, describing atomic repulsion due to the radiation pressure of scattered photons on nearby atoms, given by^41^

10 $$\begin{aligned} \mathbf {\nabla } \cdot \mathbf {F}_{R}=\frac{\sigma _{R} \sigma _{L} I_{0}}{c} n(\mathbf {r}), \end{aligned}$$

where $\sigma _R$ is the atom scattering cross section. Combining Eqs. (9) and (10), one arrives at the equation describing the collective force $\mathbf {F}_{c}=\mathbf {F}_{A}+\mathbf {F}_{R} \equiv - \nabla V_c$ or, equivalently, the associated potential $V_c$, as follows

11 $$\begin{aligned} {\Delta } V_c =-Q n(\mathbf {r}), \quad Q=\left( \sigma _{R}-\sigma _{L}\right) \sigma _{L} I_{0} / c, \end{aligned}$$

with *Q* playing the role of an effective charge (as first noticed in^30^). In typical experimental conditions, one has^30,42^ $\sigma _R > \sigma _L$, and the effective charge *Q* is positive. The system then behaves as a plasma as one can define a typical frequency

12 $$\begin{aligned} \omega _{0}=\sqrt{\frac{Q n_{0}}{m}}, \end{aligned}$$

($n_0$ being the unperturbed density), which is a straightforward generalization of the electron plasma frequency. For $\sigma _R < \sigma _L$, which may occur in a quasi 1D configuration^5^, the effective charge *Q* becomes negative and the typical frequency becomes imaginary, indicating an unstable mode; the system then behaves as a self-gravitating medium. Hence, atomic clouds trapped in MOTs can be regarded as an intermediate case between the two scenarios and can mimic the dispersion properties of both media, as detailed next. This offers new avenues for probing gravitational models in condensed-matter analog systems.

In what follows, we will show how a generic Schrödinger–Poisson equation can be constructed for gradient fields and used to unveil the signature of internal length scale parameters on the elementary excitations taking place in these media. For simplicity, we restrict ourselves in the main text to first gradient theory, that is for $\ell _2^4\rightarrow 0$, in which case Eq. (4) reduces to

13 $$\begin{aligned} L(\Delta )=1-\ell ^{2} \Delta , \end{aligned}$$

involving a single length scale parameter $\ell _1 \equiv \ell$. This is a good approximation of second gradient theory^13^ and appears as well as a limit of non-local theories of exponential type^13,43,44^, where one has

14 $$\begin{aligned} L(\Delta )=\mathrm {e}^{-\ell ^{2} \Delta }=\sum _{n=0}^{\infty } \frac{(-1)^{n}}{n !} \ell ^{2 n} \Delta ^{n}. \end{aligned}$$

The case of second gradient theory, involving two characteristic length scale parameters, can be studied following the same lines of reasoning and will be discussed in the supplementary material..

## Schrödinger–Poisson systems for gradient fields

The SP equation results from the coupling of the Schrödinger equation, for a quantum particle in the non-relativistic regime, and the Poisson equation describing the self-gravitating potential $\Phi$. That is,

15 $$\begin{aligned}&\mathrm {i} \hbar \frac{\partial \psi }{\partial t}=\left( -\frac{\hbar ^{2}}{2 m} \Delta +m \Phi \right) \psi , \\&\Delta \Phi =4 \pi G m n = 4 \pi m G|\psi |^{2}, \end{aligned}$$

where $n \equiv |\psi |^{2}$ is the (number) density. The two equations in (15) can be combined in a single integro-differential equation as follows

16 $$\begin{aligned} \mathrm {i} \hbar \frac{\partial \psi }{\partial t}=\left[ -\frac{\hbar ^{2}}{2 m} \Delta -m^{2} G \int \frac{\left| \psi \left( \mathbf {r}^{\prime }, t\right) \right| ^{2}}{\left| \mathbf {r}-\mathbf {r}^{\prime }\right| } d\mathbf {r}^{\prime }\right] \psi . \end{aligned}$$

By considering the (first) gradient modification of the Poisson equation (3), combined to the Schrödinger equation, one obtains (see supplementary material. the following integro-differential equation

17 $$\begin{aligned} \mathrm {i} \hbar \frac{\partial \psi }{\partial t}=\left[ -\frac{\hbar ^{2}}{2 m} \Delta - \frac{G m^2}{4 \pi \ell ^2} \int \frac{d \mathbf {r}''}{\left| \mathbf {r}-\mathbf {r}'' \right| } \int \frac{{\text {e}}^{-\left| \mathbf {r}''-\mathbf {r}^{\prime } \right| / \ell } \left| \psi \left( \mathbf {r}^{\prime }, t\right) \right| ^{2}}{\left| \mathbf {r''}-\mathbf {r}^{\prime }\right| } d\mathbf {r'} \right] \psi . \end{aligned}$$

The latter is an extension of the SP equation (16) to first gradient theory. We note in passing that the SP equation has interesting fundamental implications on the interpretation of quantum mechanics (see for instance^18,19^) as it describes how gravitation introduces a kind of wavefunction collapse; hence the modified SP equation (17) may shed new lights on the effect of an intrinsic length scale on that process. We avoid this digression here and follow a more pragmatic route. Rather, we are interested in the formal analogy that can be drawn by this equation between different media exhibiting similar excitations. By considering the two other systems discussed in “Gradient fields and Poisson-like equations”, one may write the following generic equation

18 $$\begin{aligned} \mathrm {i} \hbar \frac{\partial \psi }{\partial t}=\left[ -\frac{\hbar ^{2}}{2 m} \Delta + g \int \frac{d\mathbf {r}''}{\left| \mathbf {r}-\mathbf {r}'' \right| } \int \frac{{\text {e}}^{-\left| \mathbf {r}''-\mathbf {r}^{\prime } \right| / \ell } \left| \psi \left( \mathbf {r}^{\prime }, t\right) \right| ^{2}}{\left| \mathbf {r''}-\mathbf {r}^{\prime }\right| } d\mathbf {r'} + V_0 \right] \psi , \end{aligned}$$

where $V_0$ accounts for the possible presence of a background potential. Equation (18) covers the three scenarios discussed here: In the case of (i) a self-gravitating medium, one has $g \equiv -G m^2/4 \pi \ell ^2$ while the background potential $V_0$ can be safely ignored by invoking the so-called ‘Jeans swindle’^45^; for (ii) electrons in a quantum plasma, one has $g \equiv e^2 / 16 \pi ^2 \varepsilon _0 \ell ^2$ and $L (\Delta ) \Delta V_0=-e^2n_0/ \varepsilon _0$ accounts for the ionic background potential; and (iii) for an atomic cloud trapped in a MOT, one has $g \equiv Q / 16 \pi ^2 \ell ^2$ and $L (\Delta ) \Delta V_0=-Q n_{0}$, where $n_{0}$ is the equilibrium density imposed by some unspecified confinement force. Equation (18) enables us to study, in a very general context, the effect of a gradient field in the collective processes taking place in these media, namely Jeans oscillations/instability in the case of a SGS, electron states in a quantum plasma and hybrid-phonon modes in MOTs, as discussed in the next section.

## Quantum kinetic approach

In order to apply the (modified) SP equation to a system of *N* self-interacting particles, one has to construct transport equations. There are two possibilities for doing so: the hydrodynamic and the kinetic approaches (see for instance^46,47^ for a general discussion). In the hydrodynamic approach, one uses the so-called Madelung transformation to transform the SP equation into a set of hydrodynamic equations while in the kinetic representation, one relies on the use of Wigner functions to represent quantum states in a classical phase space. Those are the quantum analogs of the hydrodynamic and kinetic approaches to classical systems of *N* self-interacting particles. We follow here a kinetic treatment while we discuss the hydrodynamic formulation in “Hydrodynamics and generalised Lane–Emden equations”.

A kinetic approach, similar to the one provided by the Boltzmann equation, is possible in the quantum regime, by relying on the Wigner function, which allows representing quantum systems in a classical phase space. The Wigner function can be defined as

19 $$\begin{aligned} \begin{aligned} W(\mathbf {r}, \mathbf {p}; t) = \frac{1}{(2 \pi \hbar )^3} \int d \mathbf {y} \exp (i \mathbf {p}. \mathbf {y} / \hbar ^3)\psi ^{*}(\mathbf {r}+\mathbf {y} / 2, t) \times \psi (\mathbf {r}-\mathbf {y} / 2, t). \end{aligned} \end{aligned}$$

It is simply the Fourier transform of the autocorrelation function corresponding to the wave function $\psi$. It should be noted that the Wigner function is not a *bona fide* distribution, since it can take negative values, and should be rather regarded as a quasi-distribution. It is nonetheless a very useful mathematical tool, especially well suited for understanding the quantum/classical transition^48,49^ and collective phenomena in a variety of quantum systems^35,36,50,51^. The Wigner function (19) is normalized here such that

20 $$\begin{aligned} \int d \mathbf {p} W(\mathbf {r}, \mathbf {p}; t)=\left| \psi (\mathbf {r}, t)\right| ^{2} =n(\mathbf {r},t). \end{aligned}$$

To apply the Wigner approach to the SP equation in the context of gradient fields, instead of the integro-differential equation (18), it is more convenient to work with the coupled system composed of the Schrödinger equation and the (modified) Poisson equation. By applying the so-called Wigner–Moyal^37,38^ procedure, the system reduces to (see for instance^52^ for detailed calculations)

21 $$\begin{aligned} \begin{aligned}{}&\left( \frac{\partial }{\partial t}+\frac{\mathbf {p}}{m} \frac{\partial }{\partial \mathbf {r}}\right) W-\frac{2V}{\hbar } \sin \left( \frac{\hbar }{2} \frac{\overleftarrow{\partial }}{\partial \mathbf {r}} \frac{\overrightarrow{\partial }}{\partial \mathbf {p}}\right) W=0, \\&L(\Delta ) \Delta V= \mathscr {G} \int d \mathbf {p} W(\mathbf {r}, \mathbf {p}; t) + A, \end{aligned} \end{aligned}$$

where the sine operator is defined in terms of its Taylor expansion and the arrows indicate the sense according to which the operators act. Above, *V* stands for the (generic) potential energy term, that is (1) $V \equiv m \Phi$ for a self-gravitating system, (2) $V \equiv -e \phi$ for electrons in a plasma, and (3) $V \equiv V_c$ (viz. Eq. (11)) for MOTs. Besides, we have defined (i) $\mathscr {G} = 4 \pi G m^2$ and $A=0$ for a self-gravitating medium, (ii) $\mathscr {G}\equiv -e^2/ \varepsilon _0$ and $A=e^2 n_0/ \varepsilon _0$ for electrons in a quantum plasma, and (iii) $\mathscr {G} \equiv -Q$ and $A=Q n_{0}$ for atomic clouds in MOTs. We restrict ourselves here to the linear regime and follow the standard approach to derive the dispersion relations (see for instance^52,53^ for technical details); we introduce small perturbations around the stationary and spatially homogeneous equilibrium, given by the equilibrium distribution $W_0(\mathbf {p})$ and a constant background potential $V_0$, and express the perturbations in Fourier modes with frequency $\omega$ and wave vector $\mathbf {k}$. That is,

22 $$\begin{aligned} \begin{aligned} W(\mathbf{r} , \mathbf{p} ; t)&\equiv W_{0}(\mathbf{p} )+\tilde{W} \exp [i(\mathbf{k} . \mathbf{r} -\omega t)] \\ V(\mathbf{r} , t)&\equiv V_0+ \tilde{V} \exp [i(\mathbf{k} .\mathbf{r} -\omega t)], \end{aligned} \end{aligned}$$

with $|\tilde{W}| \ll \left| W_{0}\right|$. By linearizing Eq. (21), we obtain (In the case of a self-gravitating medium, this procedure is supplemented by invoking the so-called “Jeans swindle”, i.e., by considering that the gravitational potential is sourced only by the density perturbations and not by the density background $n_0$ (see for instance^45^ for more details).)

23 $$\begin{aligned} 1+ \ell ^2 k^2-\frac{2 i m \mathscr {G}}{\hbar k^{3}} \int _{-\infty }^{+\infty } \frac{\mathrm {d} p}{p-m \omega / k} \sin \left( \frac{i \hbar k}{2} \frac{\mathbf {\partial }}{\partial p}\right) {G_{0}}, \end{aligned}$$

where $G_0(p)$ is the projected (marginal) distribution along the axis parallel to the wave-vector $\mathbf {k}$. That is,

24 $$\begin{aligned} G_0(p)=\int W_{0}\left( p, \mathbf {p_{\perp }} \right) {d \mathbf {p_{\perp }}}, \end{aligned}$$

where *p* and $\mathbf {p_{\perp }}$ stand for the parallel and perpendicular components of the momentum respectively, i.e.,

25 $$\begin{aligned} \mathbf {p}=p \frac{\mathbf {k}}{k}+\mathbf {p}_{\perp }. \end{aligned}$$

Upon observing that

26 $$\begin{aligned} \begin{aligned} 2 \sin \left( \frac{i \hbar k}{2} \frac{\mathbf {\partial }}{\partial p}\right) {G_{0}(p)}= i\left[ G_{0}(p+\hbar k / 2)-G_{0}(p-\hbar k / 2)\right] , \end{aligned} \end{aligned}$$

Equation (23) simplifies to the following integral dispersion relation

27 $$\begin{aligned} 1+ \ell ^2 k^2 + \frac{\mathscr {G} m}{\hbar k^3}\int _{-\infty }^{\infty } \frac{G_0^+-G_0^-}{p-m \omega /k} d {p}=0, \end{aligned}$$

where $G_0^{\pm } \equiv G_{0}(p \pm \hbar k / 2)$. Equation (27) is a generic dispersion relation that remains valid for first gradient fields in the three scenarios discussed above and for arbitrary equilibrium distributions $G_0({p})$. One may check that, in the limit $\ell \rightarrow 0$, it reduces to the standard dispersion relation (see for instance^47^ for plasma oscillations and^51^ for gravitational systems). It is interesting to study the zero-temperature limit, in which case there is no dispersion in the momentum (velocity) space and the distribution $G_0({p})$ shrinks to a Dirac delta. That is,

28 $$\begin{aligned} G_{0}(p)=n_{0} \delta \left( p\right) . \end{aligned}$$

In this case, after simple algebraic manipulations, the generic dispersion relation (27) reduces to

29 $$\begin{aligned} \omega ^2= - \frac{\mathscr {G} n_0}{m (1+ \ell ^2 k^2)}+ \frac{\hbar ^2 k^4}{4 m^2}. \end{aligned}$$

In the case of a SGS ($\mathscr {G} = 4 \pi G m^2$), it reads as

30 $$\begin{aligned} \omega ^2= - \frac{ \Omega _J^2}{ (1+ \ell ^2 k^2)}+ \frac{\hbar ^2 k^4}{4 m^2}, \end{aligned}$$

where $\Omega _J \equiv \sqrt{ 4 \pi G \rho _0}$ is the so-called *Jeans frequency*. In the case of electrons in a quantum plasma ($\mathscr {G}\equiv -e^2/ \varepsilon _0$), one has

31 $$\begin{aligned} \omega ^2= \frac{ \Omega _p^2}{ (1+ \ell ^2 k^2)}+ \frac{\hbar ^2 k^4}{4 m^2}, \end{aligned}$$

where $\Omega _p \equiv \sqrt{e^2 n_0/m \varepsilon _0}$ is the electron plasma frequency. In the case of cold atomic gases in MOTs, the same equation holds for $Q>0$ with a characteristic frequency $\Omega _0 \equiv \sqrt{Q/m}$, while for $Q<0$, one has formally Eq. (30), with $\Omega _J^2 \rightarrow Q n_0/m$. In this sense, MOTs can be regarded as an intermediate case between SGSs and plasmas, regarding their dispersion properties.

**Figure 1:**
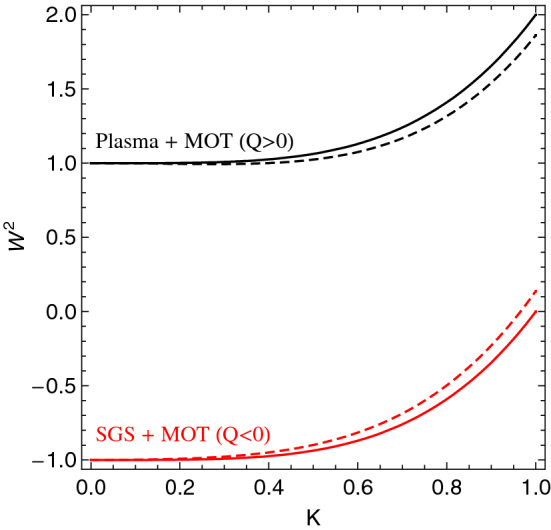
Dimensionless dispersion relations (32), covering the three scenarios of SGSs, plasmas, and MOTs. Solid lines correspond to $\mathscr{L}=0$ while dashed lines represent $\mathscr{L}=0.2$

The above dispersion relations can be written in a dimensionless form as

32 $$\begin{aligned} W^2= \frac{\pm 1}{1+\mathscr{L}^2 K^2}+K^4 \end{aligned}$$

where $W \equiv \omega / \Omega _0$ ($\Omega _0$ being the characteristic frequency of the medium), $K \equiv \lambda k$, and $\mathscr{L} \equiv \ell / \lambda$, with $\lambda \equiv \sqrt{\hbar / 2m \Omega _0}$. In Eq. (32), the (+) sign corresponds to plasmas and MOTs for $Q>0$ while the (−) sign corresponds to SGSs and MOTs with $Q<0$. The generic dispersion relation (32) is depicted in Fig. 1, showing the effect of gradient fields in the dispersion properties of these three media.

The dispersion relation for a SGS is worthy of a closer examination. In fact, because of the (−) sign in front of the first term in Eq. (30) (or Eq. 32), the frequency $\omega$ may become imaginary for small wave-numbers *k*, leading to unstable modes, i.e., *Jeans instability*. More precisely, there is a critical wave-number, or equivalently a critical wavelength, such that for perturbations with wavelengths smaller than this critical wavelength, $\omega$ is real and the perturbations correspond to sound modes, i.e., $\sim \exp i \omega t$, whereas for perturbations with wavelengths larger than the critical wavelength, $\omega$ becomes imaginary ($\omega = i \gamma$), producing two modes $\sim \exp \pm \gamma t$, one of them growing with time yielding a gravitational collapse.

In the absence of an intrinsic length scale parameter ($\ell =0$), Eq. (30) simply translates the fact that quantum pressure forces (proportional to $\hbar ^2$) act against gravity, saturating the instability for large wave-numbers. In this case, the critical wave-number, delimiting between stable and unstable modes, follows as

33 $$\begin{aligned} k_J= \sqrt{\frac{2m \Omega _J}{\hbar }}= \sqrt{\frac{8 \pi m G \rho _0}{\hbar }}. \end{aligned}$$

For $\ell \ne 0$, one obtains, by setting $\omega ^2=0$ in Eq. (30), the critical wave-number $k_{*}$. In a dimensionless form, one has

34 $$\begin{aligned} K_{*} \equiv \frac{k_{*}}{k_{J}} = \sqrt{\frac{1}{3 \mathscr{L}^2}\left( -1+\frac{1}{\xi }+\xi \right) }, \end{aligned}$$

where

35 $$\begin{aligned} \xi \equiv \left( \frac{-2+27 \mathscr{L}^4+ 3 \sqrt{3} \sqrt{-4 \mathscr{L}^4+27 \mathscr{L}^8}}{2} \right) ^{1/3}. \end{aligned}$$

**Figure 2:**
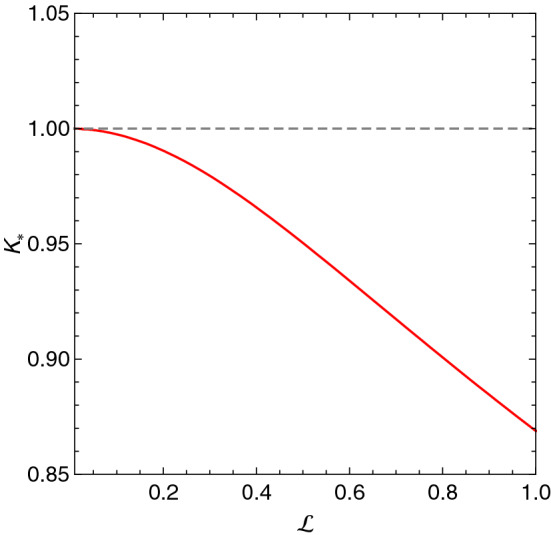
The dimensionless critical wave-number (34) as a function of the dimensionless intrinsic length scale parameter $\mathscr{L}$

Figure 2 shows the (dimensionless) wave-number (34) as a function of the dimensionless internal length scale parameter $\mathscr{L}$. It shows that, in gradient gravity, SGSs remain stable for smaller (larger) wave-numbers (wavelengths); a feature that may be attributed to the regularisation, introduced by gradient gravity, on the gravitational interactions at small length scales.

It may be interesting to compare the dispersion relations produced by gradient fields with the case of a pure Yukawa potential,

36 $$\begin{aligned} V \propto \frac{1}{\left| \mathbf {r}-\mathbf {r}^{\prime }\right| } \exp \left[ -\left( \mathbf {r}-\mathbf {r}^{\prime }\right) / \Lambda \right] \end{aligned}$$

which has been used to promote alternative theories of gravitation^54^ and occurs naturally in plasma physics^55,56^. In this case, the zero-temperature dispersion relation reads as (see^57^ for the special case of a SGS)

37 $$\begin{aligned} W^{2}=\frac{\pm K^{2} L^2}{ \left( K^{2} L^2+1 \right) }+ K^4, \end{aligned}$$

with $L \equiv \Lambda / \lambda$. In the case of a SGS, Yukawa-type gravity implies stability for larger wavelengths perturbations. More precisely, the (dimensionless) critical wave-number reads in this case as

38 $$\begin{aligned} K_*= \left( {\frac{-1+\sqrt{1+4 L^2}}{2 L^2}}\right) ^{1/2}, \end{aligned}$$

which reduces to unity in the limit $L \rightarrow \infty$. The main difference between Eqs. (37) and (32) is that the effect of the Yukawa potential appears in the long wavelength limit while the effect of gradient fields is dominant for short wavelengths.

A natural question is whether the deviations produced by gradient fields in the dispersion relations can be measured experimentally. From the generic dispersion relation (32), it is clear that such a deviation is significant only if the characteristic length of the medium $\lambda$ is of the same order of magnitude as $\ell$. In the case of a SGS, this condition is hardly conceivable since the characteristic length (essentially the Jeans length) takes astronomical values. In the two other media discussed here, however, the characteristic length $\lambda$ can be in the micrometer–millimetre range and a measurable effect with today spectroscopic methods is perfectly conceivable, especially in plasma-based experiments. In fact, in plasma-based experiments, one relies on the use of inelastic light scattering (ILS), which proved to be a powerful method for the investigation of collective excitations of a plasma, because it enables measuring energy and wave-vector dispersion of the excitations^58^. Such measurements have been pioneered by Egeler et al.^59^ and Goñi et al.^60^ who measured plasmon dispersion in GaAs quantum wire structures, using resonant ILS techniques. In our case, for such a technique to unveil a deviation from the usual dispersion relation, due to gradient fields, the characteristic length of the medium $\lambda$ (the Debye length for a plasma) has to be of the same order of magnitude as $\ell$, which requires extremely dense plasma conditions. Given the impressive development in the field of short pulse petawatt laser technology, such plasma conditions can be achieved by intense laser pulse compression using powerful X-ray pulses^61,62^. In fact, recently, spectrally resolved X-ray scattering measurements have been performed in dense plasmas, enabling for accurate measurements of plasmons in the dense matter regime^63^. To give a numerical estimate, for a typical discharge plasma, one has $n_0 \sim 10^9$–$10^{10}\,\text {cm}^{-3}$ and the characteristic length is of the order of the micrometer. For Tokamak ($n_0 \sim 10^{14}\,\text {cm}^{-3}$), we estimate $\lambda \sim 10^{-7}$ m, while for a solid-state plasma ($n_0 \sim 10^{18}\,\text {cm}^{-3}$), we estimate $\lambda \sim 10^{-9}$ m. Hence, a modification in the fields, at this scale, is potentially detectable in today plasma-based experiments. This is also potentially feasible in the case of MOTs for $Q>0$. The experimental realisation of MOTs with $Q<0$, however, remains a new field but experiments in a quasi 1D configuration with a pancake geometry, as discussed in^5^, are expected to be realised in the near future, and may open up new prospects for future laboratory experiments.

Before closing this section, it should be recalled that the results presented so far correspond to the zero-temperature limit, i.e., to a Dirac delta distribution (viz. Eq. (28)). To address finite-temperature effects, we assume an *even* distribution $G_0(p)$, which is characteristic for equilibrium and nearly equilibrium situations, and consider small quantum effects ($\hbar k/2 \ll p$). In this case, one can Taylor expand the functions $W_0^{\pm }$ and writes the integral in the dispersion relation (29) as

39 $$\begin{aligned} \int \frac{G_0^+-G_0^-}{p-m \omega /k} d {p} \approx \hbar k \int \frac{G_0'(p) dp}{p-m \omega /k} + \frac{\hbar ^3 k^3}{24} \int \frac{G_0''(p) dp}{p-m \omega /k}. \end{aligned}$$

We consider the limit of long wavelengths ($p \gg m \omega / k$), so that the singularity in the denominator is avoided. By performing a Taylor expansion of $(p-m \omega / k)^{-1}$ and keeping only terms with small values of *k* (long wavelength), one obtains the following dispersion relation

40 $$\begin{aligned} \omega ^2 \approx - \frac{\mathscr {G} n_0}{m (1+ \ell ^2 k^2)}+ 3 \frac{\langle {p^2}\rangle }{m^2} k^2+ \frac{\hbar ^2 k^4}{4 m^2}. \end{aligned}$$

The latter generalises Eq. (29) and remains valid as long as quantum effects are small. It shows that, in addition to quantum effects, thermal effects also induce a dispersion term. In the case of a SGS, this dispersion term acts, together with quantum pressure effects, to saturate the gravitational instability. Note that for a Maxwell–Boltzmann distribution (see next section), one has $\langle p^2\rangle / m^2 = k_B T /m$ while for a Fermi–Dirac distribution, one has (in the fully degenerate case) $\langle p^2\rangle / m^2 = k_B T_F / 5m$, where $T_F$ is the Fermi temperature.

## Classical limit: the Vlasov–Poisson regime

It is worth studying in more depth the classical limit of the Wigner kinetic approach. In this limit, the system of equations (21) reduces to a (modified) Vlasov–Poisson system, describing the evolution of the classical phase space distribution $f(\mathbf {r}, \mathbf {v}; t)$ under the generic potential *V*. That is

41 $$\begin{aligned} \begin{aligned}{}&\frac{\partial f(\mathbf {r}, \mathbf {v}; t)}{\partial t}+\mathbf {v} \cdot \nabla f(\mathbf {r}, \mathbf {v}; t) - \frac{\mathbf {\nabla } V}{m} \cdot \frac{\partial f(\mathbf {r}, \mathbf {v}; t)}{\partial \mathbf {v}}=0, \\&L(\Delta ) \Delta V= \mathscr {G} \int d \mathbf {v} f(\mathbf {r}, \mathbf {v}; t) + A, \end{aligned} \end{aligned}$$

where we have preferred working with the velocity $\mathbf {v}$ instead of the momentum $\mathbf {p}=m \mathbf {v}$. Following the same lines of reasoning presented in the previous section, i.e., linearising the set of equations (41) and performing a Fourier transform, we arrive at the following generic dispersion relation

42 $$\begin{aligned} 1+\ell _{}^{2} \mathbf {k}^2 +\frac{\mathscr {G}}{m \mathbf {k}^{2}} \int \frac{\mathbf {k} \cdot \frac{\partial f_{0}}{\partial \mathbf {v}}}{\mathbf {v} \cdot \mathbf {k}-\omega } d \mathbf {v}=0, \end{aligned}$$

where $f_0(\mathbf {v})$ is the equilibrium distribution function. Eq. (42) is the classical counterpart of the dispersion relation (27), and can be recovered from it by taking the formal limit $\hbar \rightarrow 0$.

It should be noted that, because of the singularity in the denominator, the integral in Eq. (42) is notoriously challenging. This singularity induces an imaginary contribution to the frequency $\omega$ which can be addressed by making the analytic continuation of the integral over *v*, along the real axis, which passes under the pole at $v=\omega /k$. In the long wavelength limit ($v \ll \omega / k$) however, the singularity is avoided. By Taylor expanding $(v- \omega /k)^{-1}$, one obtains the following (dimensionless) dispersion relation

43 $$\begin{aligned} W^2 = \frac{\pm 1}{1+ \mathscr{L}^2 K^2}+3K^2, \end{aligned}$$

where $K \equiv \lambda k$ and $\mathscr{L} \equiv \ell / \lambda$, with $\lambda \equiv \sqrt{\langle v^2 \rangle / \Omega ^2}$ (the latter corresponds to the Debye length in the case of a plasma and to the Jeans length in the case of a SGS). Equation (43) is the classical finite-temperature analogue of the quantum dispersion relation (32). As in its quantum counterpart, the (+) sign refers to plasmas while the (−) sign refers to SGSs. Cold atoms in MOTs have to be discarded here as, in this case, quantum effects always dominate over thermal effects.

In the case of a plasma, Eq. (43) represents a gradient field generalisation of the Bohm–Gross dispersion relation^64^ for electron oscillations in a classical plasma, valid in the long wavelength limit. In general, the singularity in Eq. (42) induces an imaginary contribution to the frequency $\omega$, responsible for the phenomenon of Landau damping. One can however easily check that the internal length $\ell$ does not affect this process. In fact, by noting the dispersion relation (42) as $D(k, \omega )=0$ where $D(k, \omega )$ is known as the *dielectric function*, the real and imaginary parts of the dispersion relation (42) read, respectively, as

44 $$\begin{aligned}& D_{r}\left( k, \omega _{r}\right) =1+ \ell ^2 k^2-\frac{\mathscr {G}}{m k^{2}} \mathrm {PV} \int \frac{\partial f_0 / \partial v}{v-{\omega _{r}}/{k}} d v, \\& D_{i}\left( k, \omega _{r}\right) =-\pi \left( \frac{\mathscr {G}}{m k^{2}}\right) \left[ \frac{\partial f_0}{\partial v} \right] _{v={\omega _{r}}/{k}}, \end{aligned}$$

where $PV \int$ denotes the Cauchy principal value. Assuming that the imaginary part of the frequency $\gamma$ is small as compared to the real part $\omega _r$, one has^65^

45 $$\begin{aligned} \gamma =-\frac{D_{i}\left( k, \omega _{r}\right) }{\partial D_{r}\left( k, \omega _{r}\right) / \partial \omega _{r}}. \end{aligned}$$

The effect induced by the internal length $\ell$ appears only in the real part of the dispersion relation, thus it does not affect the process of Landau damping. This can be easily explained on physical grounds; in fact, Landau damping is a purely kinetic (rather that a dynamical) effect, and is ultimately produced by the shape of the distribution function $f_0(v)$. For a single humped distribution (viz., the *Gardner theorem*), there are more particles having velocities slightly less than the phase velocity $\omega /k$, hence gaining energy from the wave, than particles having velocities slightly greater, hence losing energy to the wave. This asymmetry is the origin of Landau damping.

**Figure 3:**
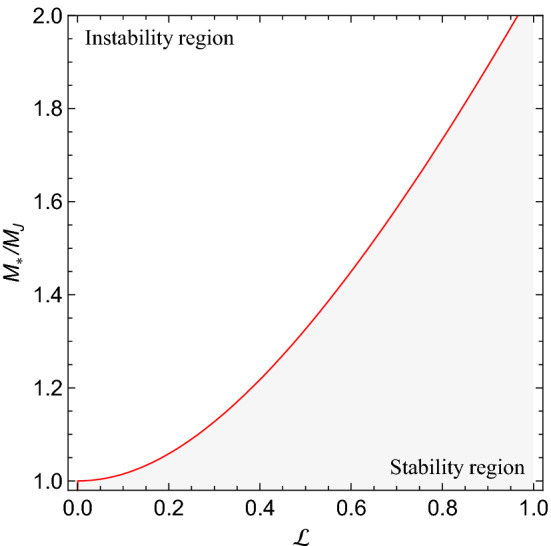
The dimensionless critical mass (48) versus the dimensionless characteristic length $\mathscr{L}$.

The case of a SGS deserves further investigation because, in this case, one has unstable modes associated with Jeans instability. By setting $W^2=0$ in Eq. (43), one may obtain the critical wave-number $k_{*}$, separating between stable and unstable modes. In a dimensionless form, one has

46 $$\begin{aligned} {K_{*}}^2 \equiv k_{*}^2/k_J^2 = \left( \frac{-1+\sqrt{1+4\mathscr{L}^2}}{2\mathscr{L}^2} \right) , \end{aligned}$$

where

47 $$\begin{aligned} k_J \equiv \sqrt{\frac{4 \pi G \rho _0}{\sigma ^2}}, \end{aligned}$$

is the standard Jeans number and $\sigma ^2 \equiv \langle v^2 \rangle$. From the critical wave-number (46), one may define the corresponding critical wavelength $\lambda _{*} \equiv 2 \pi / k_{*}$ and critical mass, i.e., the mass initially contained in a sphere of diameter $\lambda _{*}$, as

48 $$\begin{aligned} M_{*} = \left( \frac{-1+\sqrt{1+4\mathscr{L}^2}}{2\mathscr{L}^2} \right) ^{-3/2} M_J, \end{aligned}$$

where

49 $$\begin{aligned} M_{J}= \frac{4 \pi }{3} \left( \frac{\lambda _J}{2} \right) ^3 = \frac{\pi }{6} \sqrt{\frac{1}{\rho _{0}}\left( \frac{\pi \sigma ^{2}}{G}\right) ^{3}} \end{aligned}$$

is the standard Jeans mass. Figure 3 shows the critical mass $M_{*}$ as a function of the dimensionless internal length $\mathscr{L}$. One may observe that the presence of the internal length tends to saturate the instability, preventing the gravitational collapse to occur for masses larger than $M_J$.

In addition to the threshold wave-number $k_{*}$, separating between stable and unstable modes, the presence of the internal length $\ell$ also affects the growth rate of the instability. To see that, we assume a Maxwell–Boltzmann distribution

50 $$\begin{aligned} f_{0}(v)=\frac{n_{0}}{\left( 2 \pi \sigma ^{2}\right) ^{\frac{3}{2}}} e^{-\frac{v^{2}}{2 \sigma ^{2}}}, \end{aligned}$$

in which case, the dispersion relation (42) becomes

51 $$\begin{aligned} 1+\ell ^{2} {k}^2 -\frac{\mathscr {G} n_0}{m \sqrt{2 \pi } k \sigma ^{3}} \int _{-\infty }^{\infty } \frac{u e^{-\frac{u^{2}}{2 \sigma ^{2}}}}{k u-\omega } d u=0, \end{aligned}$$

where we have assumed, without loss of generality, the *x* axis to be along the direction of the wave vector $\mathbf {k}$ and have denoted $u \equiv v_x$. Equation (51) can be expressed in a dimensionless form as

52 $$\begin{aligned} \mathscr {W}(\beta )-K^2+\mathscr{L}^2 K^4 =0, \end{aligned}$$

where

53 $$\begin{aligned} \mathscr {W}(\beta ) \equiv \frac{1}{\sqrt{2 \pi }} \int \frac{x e^{-\frac{x^{2}}{2}}}{x-\beta } d x \end{aligned}$$

with $\beta := \omega /k \sigma$ and $x:=u/\sigma$. As we are concerned with unstable modes, we set $\omega = {\text {i}} \gamma$ and $\mathfrak {R}[\omega ]=0$. Upon using the identity^66^

54 $$\begin{aligned} \int _{0}^{\infty } \frac{x^{2} e^{-x^{2}}}{x^{2}+\beta ^{2}} d x=\frac{1}{2} \sqrt{\pi }-\frac{1}{2} \pi \beta e^{\beta ^{2}}[1-{\text {erf}} \beta ], \end{aligned}$$

where

55 $$\begin{aligned} {\text {erf}} z=\frac{2}{\sqrt{\pi }} \int _{0}^{z} e^{-t^{2}} d t \end{aligned}$$

is the Gauss error function, we may rewrite the dispersion relation (52) in the following form

56 $$\begin{aligned} K^{2}+\mathscr{L}^2K^4=1-\sqrt{\frac{{\pi }}{2}} \frac{ \Gamma }{K} e^{\left( \frac{\Gamma }{\sqrt{2} K }\right) ^{2}}\left[ 1-{\text {erf}}\left( \frac{\Gamma }{\sqrt{2} K}\right) \right] \end{aligned}$$

where $\Gamma := \gamma / \Omega _J$ is the dimensionless growth rate of the instability. We have solved numerically Eq. (56). As shown in Fig. 4, the presence of the internal length scale $\ell$ reduces also the growth rate of the instability.

**Figure 4:**
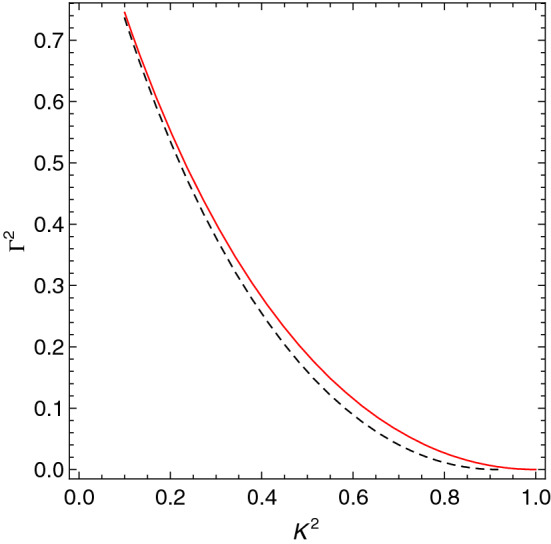
The growth rate corresponding to the numerical solution of Eq. (56) for Newtonian gravity (solid line) and first gradient gravity with $\mathscr{L}=0.1$ (dashed line).

## Hydrodynamics and generalised Lane–Emden equations

In addition to the kinetic approach, discussed above, the other route for studying collective phenomena consists in the hydrodynamic approach. Although the latter is less accurate than the former, it may be easier to implement in numerical simulations and has the advantage of making more explicit the interplay between the (gravitational or electrostatic) interactions and pressure forces. In the classical context, the set of hydrodynamic equations can be obtained by taking the moments of the Vlasov equation (viz. the first equation in Eq. (41)). The equations governing the evolution of the zeroth order (i.e., the density $n(\mathbf {r})$) and first order (i.e., the velocity field $\mathbf {u}(\mathbf {r})$) moments, follow from the Vlasov equation as

57 $$\begin{aligned} & \frac{\partial n}{\partial t}+\mathbf {\nabla } \cdot (n \mathbf {u})=0, \\& m \left( \frac{\partial \mathbf {u}}{\partial t}+\mathbf {u} \cdot \mathbf {\nabla } \mathbf {u} \right) =-\frac{\mathbf {\nabla } P}{ n}-\mathbf {\nabla V}, \end{aligned}$$

where *V* is a generic potential. The first equation in (57) is the continuity equation while the second one is the Euler (momentum balance) equation. In *hydrostatic equilibrium*, the interactions are counterbalanced by the pressure force, that is

58 $$\begin{aligned} \nabla \cdot \left[ \frac{\nabla P}{n} \right] =- \Delta V. \end{aligned}$$

Assuming a polytropic equation of state, one may derive, from the condition of hydrostatic equilibrium (63) and the Poisson equation, the Lane–Emden equation, allowing to study self-gravitating spheres^67^. Likewise, a Lande–Emden equation can also be formally derived for other media, such as plasmas, MOTs, and Bose-Einstein condensates^68–70^. In this section, we derive a generalised Lane–Emden equation, in the presence of gradient fields, that remains valid for SGSs, plasmas, and MOTs. We assume a polytropic equation of state

59 $$\begin{aligned} P=C_{\gamma } n^{\gamma }, \end{aligned}$$

where $C_{\gamma }=P_0 / n_0^{\gamma }$ is a constant depending on the conditions of the thermodynamic transformation, and $P_0$ and $n_0$ represent, respectively, the pressure and the density at the centre of the cloud. The *polytropic exponent* $\gamma$ is related to the *polytropic index* $\alpha$ through

60 $$\begin{aligned} \gamma = 1+ \frac{1}{\alpha } \quad \Leftrightarrow \quad \alpha = \frac{1}{\gamma -1}. \end{aligned}$$

We assume spherical symmetry and express the density as $n({r}) \equiv n_0 \theta ({r})^{\alpha }$. By inserting the polytropic equation of state (59) in Eq. (63) and making use of the generalised Poisson equation, we obtain the following generalisation of the Lane–Emden equation

61 $$\begin{aligned} \left( \frac{2}{\xi } \frac{d \theta }{d \xi }+\frac{d^{2} \theta }{d \xi ^{2}}\right) - \mathscr{L}^2 \left( \frac{4}{\xi } \frac{d^{3} \theta }{d \xi ^{3}}+\frac{d^{4} \theta }{d \xi ^{4}}\right) =-\theta ^{\alpha }, \end{aligned}$$

where we have defined the dimensionless variable $\xi \equiv r / \lambda$, and $\mathscr{L} \equiv \ell / \lambda$, with the characteristic length defined as

62 $$\begin{aligned} \lambda \equiv \sqrt{\frac{(1+\alpha ) C_{\gamma } n_0^{\frac{1-\alpha }{\alpha }}}{ \mid \mathscr {G} \mid }}. \end{aligned}$$

We recall that we have (1) $\mathscr {G} = 4 \pi G m^2$ for a SGS, (2) $\mathscr {G}\equiv -e^2/ \varepsilon _0$ for electrons in a quantum plasma, and (3) $\mathscr {G} \equiv -Q$ for atomic clouds in MOTs. Equation (61) is a generalisation of the Lane–Emden equation in the presence of gradient fields, and remains valid in the three physical scenarios discussed here, namely, SGSs, plasmas, and MOTs. In the absence of an internal length scale parameter, $\mathscr{L}=0$, it reduces to the standard Lane–Emden equation^67^.

**Figure 5:**
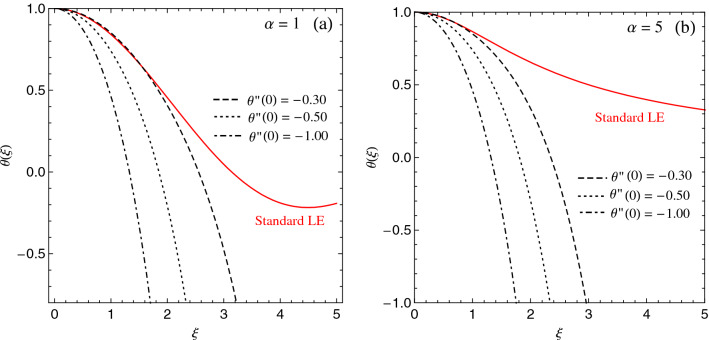
Numerical solutions of the generalized Lane–Emden equation (61) for (**a**) $\alpha =1$ and (**b**) $\alpha =5$, with $\mathscr{L}^2=0.5$ and different values of $\theta ''(0)$.

A word of caution on the applicability of Eq. (61) to draw physically meaningful conclusions is however in order here. It should be noted that the standard Lane–Emden equation is a differential equation of second order. Physically relevant solutions are those satisfying $\theta (0)=1$ and $\theta '(0)=0$^67^, which amounts to fix the density at the centre of the cloud to $n_0$, as an extremum (typically, a peak density). Finding physically relevant solutions to the higher-order generalised Lane–Emder equation (61) requires extra-information on the boundary conditions. In typical situations, the density is maximum at the centre, which amounts to impose $\theta (0)''<0$. Figure 5 shows examples of numerical solutions of Eq. (61) for two polytropic indices, namely $\alpha =0$ and $\alpha =5$, and different conditions on $\theta ''(0)$, together with the analytic solution of the standard Lane-Emden equation, which is analytically solvable for these polytropic indices.

It should be also noted that equation (61) has been derived here in the classical regime. Quantum effects can nonetheless be incorporated in the generalised Lane–Emden equation (61) by accounting for the quantum pressure force in the condition of hydrostatic equilibrium (63). That is,

63 $$\begin{aligned} \nabla \cdot \left[ \frac{\nabla P}{n} \right] - \frac{\hbar ^2}{2m} \Delta \left( \frac{\Delta \sqrt{n}}{\sqrt{n}} \right) =- \Delta V. \end{aligned}$$

The effect produced by the quantum pressure force is however negligibly small and can be safely neglected. Likewise, in the presence of an external potential, for example a trapping potential in the case of MOTs, the latter has to be accounted for in the condition of hydrostatic equilibrium, and will contribute through an extra term in the generalised Lane–Emden equation (61) (see for instance^68^).

## Conclusion and outlook

We have addressed, in this paper, the imprints of gradient field theory—a procedure allowing for a straightforward regularisation of the fields^13–17^—in the dispersion properties of various media. To put the discussion on very general grounds, we considered the general scenario of systems obeying the Schrödinger–Poisson equation and relied on the Wigner–Moyal kinetic approach. This allowed us to treat, on equal footing, three physical situations, namely, self-gravitating systems, plasmas, and ultra-cold atomic gases in magneto-optical traps. We addressed the signature of gradient fields in the elementary excitations taking place in these media, namely Jeans oscillations/instability, electron states or plasmons, and hybrid-phonon modes. This opens new opportunities to experimentally test gradient fields, especially in plasma-based experiments where we have estimated this effect to be accessible in state-of-the-art laboratory experiments. We have discussed in more detail the classical kinetic and hydrodynamic limits of our approach and derived a class of generalised Lane–Emden equation that remain valid in these three scenarios.

This work may open up new prospects for future research, both at the experimental and the theoretical levels. At the experimental level, it may inspire future laboratory experiments to test gradient fields through their effects on elementary excitations. At the theoretical level, several generalisations of our approach seem worthy of further investigation. First, it may be interesting to go beyond the linear regime, discussed here, and seek the signature of gradient fields in the dynamics of nonlinear structures taking place in these media, such as solitons, shock waves, voids, etc. This may open up new possibilities for laboratory tests with, potentially, a higher degree of precision. Second, it may be interesting to generalise our approach to the relativistic domain. For spinless particles, the Schrödinger equation has to be replaced by the Klein–Gordon equation, and the relativistic analogue of Eq. (18) reads as

64 $$\begin{aligned} \begin{aligned} \biggl [\mathrm {i} \hbar \frac{\partial }{\partial t}-V_{0} - g \int \frac{d d\mathbf {r''}}{\left| \mathbf {r}-\mathbf {r}'' \right| } \int \frac{{\text {e}}^{-\left| \mathbf {r}''-\mathbf {r}^{\prime } \right| / \ell } \left| \psi \left( \mathbf {r}^{\prime }, t\right) \right| ^{2}}{\left| \mathbf {r''}-\mathbf {r}^{\prime }\right| } {d\mathbf {r'}} \biggl ]^{2} \psi =\left( m^{2} c^{4}-\hbar ^{2} c^{2} \Delta \right) \psi . \end{aligned} \end{aligned}$$

The latter can equally well be treated using the Wigner–Moyal approach (see for instance^71^), which enables studying the effect of gradient fields on the dispersion properties of relativistic quantum systems. This may be relevant for self-gravitating systems and plasmas. The case of cold atoms in magneto-optical traps, however, has to be discarded since it always belongs to the non-relativistic domain.

## Supplementary Information

Supplementary Information.

## Data Availability

All data generated or analysed during this study are included in this published article and its supplementary information files.
