# Supplementary material for: Schrödinger–Poisson systems under gradient fields
Source: Sci Rep. 2022 Sep 20;12:15717. doi: 10.1038/s41598-022-20107-9 (PMC9489758; doi:10.1038/s41598-022-20107-9)
Supplement: Supplementary file 1 — Supplementary Information. [file 41598_2022_20107_MOESM1_ESM.pdf]

Supplementary material for: Schrödinger-Poisson systems under gradient fields

Kamel Ourabah[1,*]

[1]Theoretical Physics Laboratory, Faculty of Physics, University of Bab-Ezzouar, USTHB, Boite Postale 32, El Alia, Algiers 16111, Algeria
[*]kam.ourabah@gmail.com

ABSTRACT

We provide here technical details not shown in the manuscript.

Derivation of Eq. (17)

We may consider, as a starting point, the screened Poisson equation

$$\left[\nabla^2-\alpha^2\right] u(\mathbf{r})=-f(\mathbf{r}), \tag{1}$$

where α is a constant and $f(\mathbf{r})$ is an arbitrary function of position. In three dimensions, equation (1) has the following solution (see e.g.,[1,2])

$$u(\mathbf{r})=\int d^3\mathbf{r}' \frac{\mathrm{e}^{-\sqrt{\alpha^2}|\mathbf{r}-\mathbf{r}'|}}{4\pi|\mathbf{r}-\mathbf{r}'|} f\left(\mathbf{r}'\right), \tag{2}$$

that is, a superposition of screened $1/\mathbf{r}$ functions, weighted by the function $f(\mathbf{r})$ and with α acting as the strength of the screening. By setting $u(\mathbf{r})=\nabla^2\Phi(\mathbf{r})$ and imposing $u(r\to\infty)=0$, one has from Eq. (3) in the manuscript

$$u(\mathbf{r})=\frac{mG}{\ell^2}\int \frac{d^3\mathbf{r}'\,\mathrm{e}^{-|\mathbf{r}-\mathbf{r}|/\ell}}{|\mathbf{r}-\mathbf{r}'|}|\psi(\mathbf{r}')|^2, \quad \ell>0 \tag{3}$$

and

$$\Phi(\mathbf{r})=-\int \frac{d^3\mathbf{r}'}{4\pi|\mathbf{r}-\mathbf{r}'|} u\left(\mathbf{r}'\right). \tag{4}$$

By combining equations (3) and (4), one obtains the self-gravitating potential

$$\Phi(\mathbf{r})=\frac{Gm}{4\pi\ell^2}\int \frac{d^3\mathbf{r}'}{|\mathbf{r}-\mathbf{r}'|}\left(\int \frac{d^3 r''\,\mathrm{e}^{-|\mathbf{r}''-r'|/\ell}}{|\mathbf{r}'-\mathbf{r}''|}|\psi(\mathbf{r}'')|^2\right) \tag{5}$$

entering into the modified SP equation [Eq. (17) in the manuscript].

Dispersion relations in second gradient field theory

While in the main text, we restrict ourselves, for simplicity, to first gradient fields, we present here the dispersion relations corresponding to second gradient fields. By keeping the second gradient term in $L(\Delta)$ [Eq. (4) in the manuscript] and following the same procedure, we obtain, in the quantum regime, the following dimensionless dispersion relation

$$W^2=\frac{\pm 1}{1+\mathscr{L}_1^2K^2+\mathscr{L}_2^2K^4}+K^4, \tag{1}$$

where $\mathscr{L}_{1,2} \equiv \ell_{1,2}/\lambda$, which is the second gradient extension to Eq. (32).

In the classical regime, following the same procedure, we have

$$W^2 = \frac{\pm 1}{1+\mathscr{L}_1^2 K^2 + \mathscr{L}_2^2 K^4} + 3K^2, \tag{2}$$

which is a second field extension of Eq. (43). Likewise, for a Maxwell-Boltzmann distribution, we have

$$K^2 + \mathscr{L}_1^2 K^4 + \mathscr{L}_2^4 K^6 = 1 - \sqrt{\frac{\pi}{2}} \frac{\Gamma}{K} e^{\left(\frac{\Gamma}{\sqrt{2}K}\right)^2} \left[1 - \mathrm{erf}\left(\frac{\Gamma}{\sqrt{2}K}\right)\right], \tag{3}$$

which is the second gradient extension to Eq. (56).

References

1. Jackson, J. D. *Classical Electrodynamics*, 3rd edn (Wiley, New York, 1998).

2. Jentschura, U. D. *Advanced Classical Electrodynamics: Green Functions, Regularizations, Multipole Decompositions* (World Scientific, Singapore, 2017).
